# High Expression of TGF-β1 Contributes to Hepatocellular Carcinoma Prognosis *via* Regulating Tumor Immunity

**DOI:** 10.3389/fonc.2022.861601

**Published:** 2022-04-25

**Authors:** Xiuli Jin, Shuairan Zhang, Ningning Wang, Lin Guan, Chuanli Shao, Yingbo Lin, Jianping Liu, Yiling Li

**Affiliations:** ^1^ Department of Gastroenterology, First Affiliated Hospital of China Medical University, Shenyang, China; ^2^ Department of Medical Oncology, First Affiliated Hospital of China Medical University, Shenyang, China; ^3^ Emergency Department, Bengbu First People’s Hospital, Bengbu, China; ^4^ Department of Oncology-Pathology, Karolinska Institute, Stockholm, Sweden; ^5^ Department of Gastroenterology, First Affiliated Hospital of Nanchang University, Nanchang, China

**Keywords:** hepatocellular carcinoma, TGF-β1, tumor-infiltrating immune cells (TICs), prognosis, survival

## Abstract

**Background:**

Transforming growth factor-beta (TGF-β) signaling is essential in initialization and progression of hepatocellular carcinoma (HCC). Therefore, a treatment targeting TGF-β pathway may be a promising option for HCC control.

**Methods:**

First, publicly available RNA-seq datasets and clinical characteristics of 374 HCC patients in The Cancer Genome Atlas (TCGA) database were downloaded. Then, Cox regression analysis and LASSO analysis were used to construct a prognostic model for TGF-β family genes. The area under the curve (AUC) of the risk signature was calculated to evaluate the predictive power of the model. Cox regression analysis was applied to predict whether TGF-β1 can be an independent prognosis factor for HCC. Next, hazard ratio and survival analyses were performed to investigate the correlation between TGF-β1 expression and survival time. Furthermore, differential expression level of TGF-β1 in HCC tissues and cells was determined. In addition, Gene Set Enrichment Analysis (GSEA) identified the top significantly activated and inhibited signal pathways related to high expression of TGF-β1. Finally, the CIBERSORT tool was adopted to correlate the tumor-infiltrating immune cells (TICs) with TGF-β1 expression in HCC cohorts.

**Results:**

Cox regression analysis and LASSO analysis revealed that seven TGF-β family members (including TGF-β1) could be used as prognostic factors for HCC. Interestingly, TGF-β1 was demonstrated to be an independent prognostic factor of HCC. RT-qPCR and immunofluorescence staining confirmed the high expression of TGF-β1 in HCC cell lines and tissues, which is significantly related to pathological classifications, poor prognosis, and short survival time. Finally, GSEA and CIBERSORT analyses suggested that TGF-β1 may interact with various immune cells and influence the prognosis of HCC patients through Tregs and γδ T cells.

**Conclusion:**

We established a novel prognostic prediction method to predict the risk scores of TGF-β genes in HCC prognosis. TGF-β1 is highly expressed in HCC cell lines and tissues, correlates to poor prognosis, and thus can be used as a potential biomarker to predict HCC prognosis. We showed that TGF-β1 may play its roles in HCC prognosis by modulating the immune microenvironment of tumor cells. Our data may shed more light on better understanding the role of TGF-β1 in HCC prognosis.

## Introduction

Hepatocellular carcinoma (HCC) is one of the most common and lethal cancers worldwide, especially in developing countries (1). Despite the development of multi-modal and advanced therapeutics, including surgical approaches and systemic drug treatment, the overall survival of HCC patients remains rather poor (2). Attributed to the in-depth study of the tumor microenvironment (TME), immunotherapy has become a promising treatment for HCC patients (3, 4). However, the complexity of TME highlights the presence of multiple non-redundant mechanisms of cancer immune-suppression, which is recognized as a critical barrier against immunotherapy (5). Consequently, identification of fundamental target genes and signal pathways related to immunotherapy in HCC is imperative for developing novel HCC therapeutic agents.

Transforming growth factor beta (TGF-β) is a family of cytokines and growth factors encoded by 32 genes, which can be divided into two groups, represented by TGF-βs and bone morphogenic proteins (BMPs) (6). TGF-β, the prototypical member of the TGF-β family, consists of three isoforms in mammals (TGF-β1, β2, and β3), among which TGF-β1 is the most abundant and well-studied family member (7). TGF-β and the TGF-β signal pathway, in orchestration with many other important genes and pathways, play central roles in various cancer initialization, progression, and treatment (8). First, TGF-β expression is significantly increased through cancer progression, which is often correlated to a poor prognosis (9). TGF-β signaling is involved in all the stages of liver disease progression from initial liver injury to development of HCC (10). Then, TGF-β can drive changes in immune cells to induce a strong immune-suppressive milieu (11). Meanwhile, TGF-β may trigger the signals in a paracrine manner to promote tumor progression in the TME (12, 13).

As a highly complicated system, the TME is composed of various cell types and molecules, including tumor cells, infiltrating immune cells, cancer-associated stromal cells, endothelial cells, and lipocytes, along with some extracellular matrix (ECM) and multiple signaling molecules (14, 15). The tumor immune microenvironment (TIME) is a novel concept that is closely related to the clinical prognosis of patients with tumors (16). The proportions of immunosuppressive cells such as M2-type macrophages, regulatory T (Treg) cells, and myeloid-derived suppressor cells (MDSCs), which are modified by CAFs (cancer-associated fibroblasts), have been shown to be significantly increased in the TIME, thereby contributing to tumor immune suppression (17–19). Accumulating evidence has shown that TGF-β signaling is involved in the regulation of immunosuppressive microenvironment (6, 20, 21).

In the present study, using various bioinformatic approaches, RT-qPCR and immunofluorescence staining techniques, we analyzed the correlation between TGF-β gene family and HCC progression, with the focus on TGF-β1. We aim to explore the possibility of utilizing TGF-β1 as a potential marker for immunotherapy and prognostic evaluation of HCC. Finally, we found that TGF-β1 is abnormally highly expressed and related to immune cells in HCC samples, which may provide new insights for early diagnosis and immunotherapy of HCC.

## Materials and Methods

### Extraction of HCC RNA-seq Data from TCGA

RNA-seq FPKM (Fragments Per Kilobase of transcript per Million mapped reads) datasets of 374 HCC patients including 50 para-cancerous samples, and the clinical information were downloaded from The Cancer Genome Atlas Liver Hepatocellular Carcinoma (TCGA-LIHC, www.portal.gdc.cancer.gov).

### Bioinformatic Tools Used in This Study

R package was installed through Rstudio (version 4.12). The following packages or software were used for downstream analyses: beeswarm (v 0.4.0), CIBERSORT (v 1.03), corrplot (v 0.92), e1071 (v 1.7-9), forestplot (v 2.0.1), ggplot (v 2.3.3.3), ggpubr (v 0.4.0), ggextra (v 0.9), glmnet (v 3.6.2), GSEA (v 4.10), limma (v 3.48.3), Parallel (stage 4), Preprocesscore (v 1.54.0), survival (v 3.2-13), survminer (v 0.4.9), tidyverse (v 1.3.1.), timeROC (v 0.4), and vioplot (v 0.3.7).

### Construction of the Prognostic Model for TGF-β-Related Genes

Thirty-two TGF-β-related genes were selected according to the previous studies (22–24). Expression data of the 32 genes were extracted and correlated with the survival information. Univariate Cox regression analyses were performed based on the pre-processed data. Hazard ratio (HR) was computed using the survival, survminer, and forestplot packages. To avoid overfitting of the model, least absolute shrinkage and selection operator (LASSO) regression was applied to reduce model dimension, and the optimal lambda values were computed for each significant gene.

### Validation of the Prognostic Model for TGF-β-Related Genes

Risk value was calculated for each HCC patient based on the coefficient using the following formula:


$$RiskScore=\sum_{i=1}^{\text{n}}coef(gene)*\exp(gene)$$

Taking the median of the risk value as a cutoff, the HCC patients were divided into high- and low-risk groups for risk score calculation and survival analysis. Receiver operating characteristic (ROC) curves for 1-, 3-, and 5-year survival time were plotted, with the area under curve (AUC) calculated to illustrate the prognostic capability of TGF-β related genes in HCC progression.

### GSEA, GO, and Immune Infiltration Analysis Based on TGF-β1 Expression

Based on the TGF-β1 expression of the HCC patients, we performed differential expression analysis, survival analysis, clinical association analysis, and prognosis analysis using multiple R packages. First, GSEA (Gene Set Enrichment Analysis) was carried out against KEGG (Kyoto Encyclopedia of Genes and Genomes) database to analyze the differential activation of signal pathways between TGF-β1 high cohort and TGF-β1 low group. NES (normalized enrichment score) >2, and *p*-value and adjusted *p*-value < 0.01 were considered as significant. Similarly, Gene Ontology (GO) enrichment was performed to compare the difference in biological processes, cellular components, and molecular functions between the two groups. |log2FoldChange| > 1 and *p* < 0.05 were considered as significant. Then, immune infiltration analysis was conducted using CIBERSORT to compute the percentage of the 22 types of immune cells in each HCC sample and correlated to its TGF-β1 expression.

### Cell Culture

Human fetal hepatocyte cell line LO2 and liver HCC cell lines (HepG2 and Huh7) were purchased from Cell Bank of Chinese Academy of Sciences and tested to be mycoplasma negative (LookOut^®^ Mycoplasma PCR Detection Kit, Cat. No.: MP0035, Sigma-Aldrich) prior to this study. After identity confirmation using Goldeneye™ 20A STR complex Amplification Kit (Goldeneye Ltd, Beijing, China), the cells were grown to approximate 80% confluence in Dulbecco’s modified Eagle’s medium with high glucose (HyClone) supplemented with 10% fetal bovine serum (FBS) (Gibco) and 1% penicillin and streptomycin at 37°C and 5% CO_2_.

### Tissues from HCC Patients

Eighteen pairs of HCC and adjacent para-cancerous tissues were collected from patients with their consent at the Department of Hepatobiliary Surgery, the First Affiliated Hospital of China Medical University between May 2018 and April 2019. The sample collection was approved by the research ethics committee of First Affiliated Hospital of China Medical University. All collected samples were immediately frozen in liquid nitrogen and transferred to a −80° freezer until further analyses.

### RNA Extraction and RT-qPCR

Total RNAs from fifteen pairs of frozen tissues and three cell lines were extracted using TRIzol reagent (Invitrogen), followed by cDNA synthesis using the PrimeScript RT Reagent Kit (TaKaRa). The SYBR Prime Script RT-PCR kit (TaKaRa) was used for RT-qPCR according to the manufacturer’s protocol. The relative expression was calculated using the ΔΔ^Ct^ method. *β-ACTIN* served as an internal control. The following two pairs of primer sets are used: TGFB1-F, 5’-CAAGCAGAGTACACACAGCAT-3’ and TGFB1-R, 5’-TGCTCCACTTTTAACTTGAGCC-3’; ACTB-F, 5’-ATGTGGCCGAGGACTTTGATT-3’ and ACTB-R, 5’-AGTGGGGTGGCTTTTAGGATG-3’.

### Immunofluorescence Staining

Each specimen was fixed with 10% formalin, dehydrated, and embedded in paraffin. Paraffin section was 4 μm thick on glass slide. Firstly, the sections were permeabilized with 100 μl of 0.5% Triton X-100 at room temperature for 10–15 min. Then, sections were washed three times with PBS (phosphate-buffered saline). Second, the sections were washed once with 1% BSA, followed by blocking with 5% BSA. Then, the sections were incubated with primary antibody against TGF-β1 (1:100; proteintech, #21898-1-AP) at 4°C overnight. Next, the sections were washed three times with PBS in a shaker, each time for 5 min. Subsequently, a secondary goat anti-rabbit antibody (1:100, proteintech, #SA00013-2) was applied to the slides at room temperature for 2 h, followed by washing three times with PBS in a shaker, each time for 5 min. Furthermore, nuclei was counter-stained using DAPI (1:1000) for 10 min, followed by washing three times with PBS in a shaker, each time for 5 min. Finally, the sections were subjected to image acquisition at confocal microscopy (Nikon, A1R).

### Statistical Analyses

The charts, forest plots, and calibration plots were visualized and statistically analyzed using SPSS (version 22.0) and R (4.12). Univariate Cox regression analysis, LASSO regression analysis, and Spearman correlation coefficient were used to calculate the risk scores for the selected TGF-β genes, as the independent prognosis factors for HCC. Survival curves were generated using the Kaplan–Meier method. Univariate and multi-variant Cox regression analyses were adopted to predict the correlation between the selected gene and survival prognosis. Statistical differences were computed by independent *t* test between two groups. *p* < 0.05 was considered statistically significant.

## Results

### TGF-β Family Genes as Prognostic Factors for HCC Progression

First, detailed clinical and follow-up information (gender, age, histological grade, pathologic stage, TNM stage, and prognosis) of the 374 HCC samples were downloaded from the TCGA platform and compiled in **Supplementary Table 1**. Higher grade (1-4) or higher stage (1-4) indicates more malignant. TNM stands for tumor node metastasis, where T (T1-T4) implies tumor size, N suggests the existence, localization, and number of lymph node, while M means metastasis.

**Figure 1:**
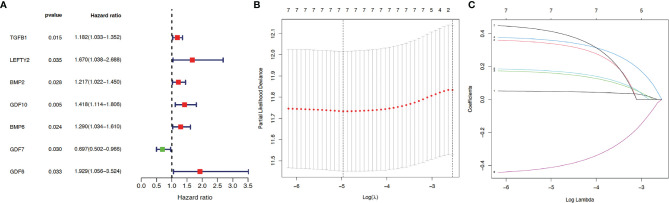
Construction of prognosis model. Thirty-two genes from the TGF-β family were subjected to univariate Cox regression analysis. **(A)** Seven TGF-β family members (including TGF-β1) were identified as independent prognostic factors for hepatocellular carcinoma (HCC) using survival, survminer, and forestplot packages. Red indicates positive correlation while green indicates negative correlation. *p* < 0.05 suggests statistical significance. LASSO regression analysis was applied to reduce model complexity and prevent over-fitting, with the optimal lambda values shown in **(B)** As indicated in **(C)**, after analysis using glmnet and survival packages, each curve in the LASSO regression represents a gene, with the coefficients shown in the *y*-axis. The numbers on the top of **(B, C)** indicate how many genes are left in the model when Lambda is set to a certain value. The coefficients from top to bottom in **(C)** correspond to the respective genes as follows: GDF6 (0.41), GDF10 (0.36), LEFTY2 (0.34), BMP6 (0.17), BMP2 (0.16), TGFB1 (0.049), and GDF7 (−0.41).

Next, to understand the risk signature of 32 TGF-β related genes in HCC progression, we analyzed the correlation between the expression levels of the 32 genes in HCC patients with HCC progression. Univariate Cox analysis (**Figure 1A**) predicted that 7 out of the analyzed 32 TGF-β related genes are significantly correlated with HCC progression (*p* < 0.05).

Then, for a more accurate prediction, LASSO regression was used to calculate the optimal lambda values (**Figure 1B**) and the coefficient for each gene (**Figure 1C**). Taken together, seven TGF-β family members (TGF-β1, LEFTY2, BMP2, BMP6, GDF6, GDF7, and GDF10) were identified as independent prognostic factors for HCC.

**Figure 2:**
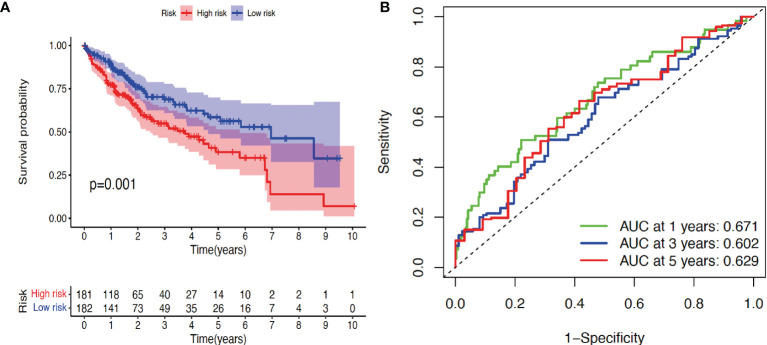
Validation of prognosis model. **(A)** Kaplan–Meier survival curve analysis of high-risk and low-risk cohorts using package survival. **(B)** Receiver operating characteristic (ROC) analysis of three different survival times (1, 3, and 5 years) using timeROC and ggplot2 packages. AUC: area under the curve. *p* < 0.05 indicates statistical significance.

Finally, according to the median risk score, the cohort of patients from TCGA database was divided into high-risk and low-risk groups (**Supplementary Figure 1A**). The high-risk group exhibited a higher frequency of poor overall survival (OS) than the low-risk group, as shown by the survival time (**Supplementary Figure 1B**) and Kaplan–Meier survival curve (**Figure 2A**). Principal component analysis (PCA) revealed that these two groups had tendency towards opposite directions (**Supplementary Figure 1C**), confirming the validity of the prediction model, which was further supported by the AUC analysis of 1-, 3-, and 5-year survival time (**Figure 2B**). Therefore, we conclude that some of the TGF-β family genes can be used as prognostic factors for HCC progression.

### TGF-β1 as a Prognostic Factor for HCC Progression

**Figure 3:**
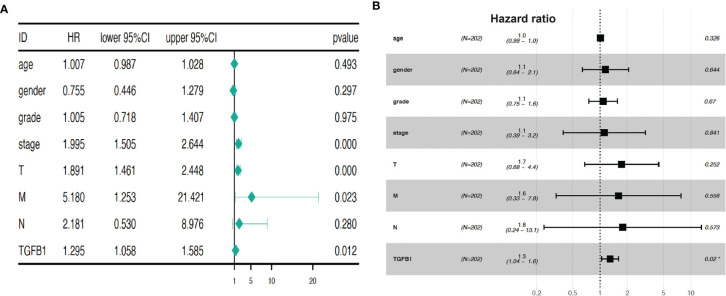
TGF-β1 expression is a prognostic factor for HCC progression. Univariate Cox regression analysis **(A)** and multivariate Cox regression **(B)** demonstrated that TGF-β1 could be a prognostic factor of HCC. Hazard ratio was computed using survival, survminer, and forestplot packages. HR, hazard ratio; CI, confidence intervals; T, tumor; M, metastasis; N, (lymph) node. *p* < 0.05, *p* < 0.01, and *p* < 0.001 suggest significance, strong significance, and extremely strong significance, respectively.

By correlating each single factor with survival time of each patient, Univariate Cox regression analysis (**Figure 3A**) showed that four factors (stage, T, M, and TGF-β1) are potential prognostic factors of clinical relevance and significance (*p* < 0.05). However, HCC prognosis is not significantly related with age, gender, grade, or node (*p* > 0.05). Furthermore, multivariate Cox regression analysis (**Figure 3B**) demonstrated that TGF-β1 could be an independent prognostic factor of HCC, with *p* = 0.02. Taken together, Cox regression analyses suggested that expression level of TGF-β1 is closely related to HCC progression.

### High TGF-β1 Expression in HCC Tissues With Poor Survival Time

**Figure 4:**
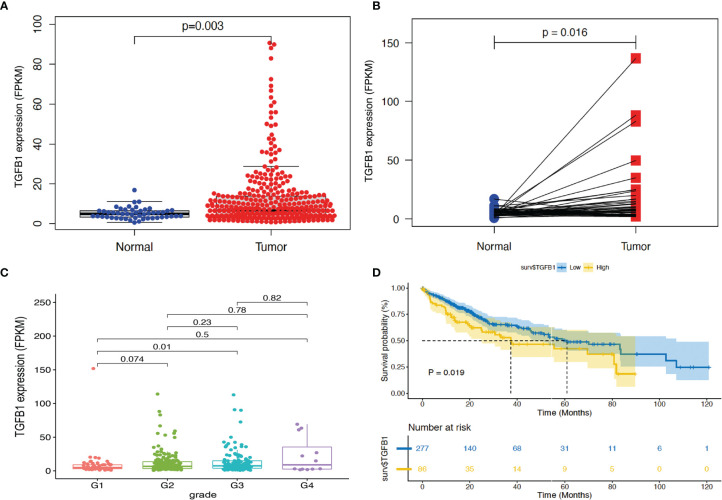
Bioinformatic analysis revealed high TGF-β1 expression in HCC tissues, with poor survival time. RNA-seq data (FPKM) of 374 HCC tissues and 50 non-HCC liver tissues were extracted from TCGA database (portal.gdc.cancer.gov). TGF-β1 expression and *p*-value were computed using limma and beeswarm packages for the 374 samples **(A)** and the 50 pairs of HCC tissues with the corresponding neighboring non-tumorous liver tissues **(B)**. **(C)** Correlation analysis of TGF-β1 expression in HCC tissues of different grades using ggpubr and tidyverse packages. G1: *N* = 55; G2: *N* = 179; G3: *N* = 124; G4: *N* = 12. **(D)** Kaplan–Meier survival curve analysis of TGF-β1 expression level and survival time using the survival package. *p* < 0.05 and *p* < 0.01 suggest significance and strong significance, respectively.

To further validate the correlation of TGF-β1 expression level with HCC prognosis, we first implemented some bioinformatic tools including limma, beeswarm, ggpubr, and tidyverse to analyze the RNA-seq data (FPKM) of the HCC tissues extracted from the TCGA database. Differential expression scatter plot (**Figure 4A**) revealed high TGF-β1 expression in HCC samples when compared with normal liver tissue samples, which was confirmed by the paired differential analysis (**Figure 4B**) using the 50 pairs of HCC tissues and the corresponding neighboring non-tumorous liver tissues.

Next, clinical correlation analysis (**Figure 4C**) showed that TGF-β1 expression increased along with the HCC progression through different grades, indicating poor differentiation and prognosis when TGF-β1 expression is high. However, only statistical significance was observed when comparing the data from grade 1 with that from grade 3. Furthermore, Kaplan–Meier survival curve analysis demonstrated that high TGF-β1 expression level is significantly correlated with poor survival time (**Figure 4D**).

**Figure 5:**
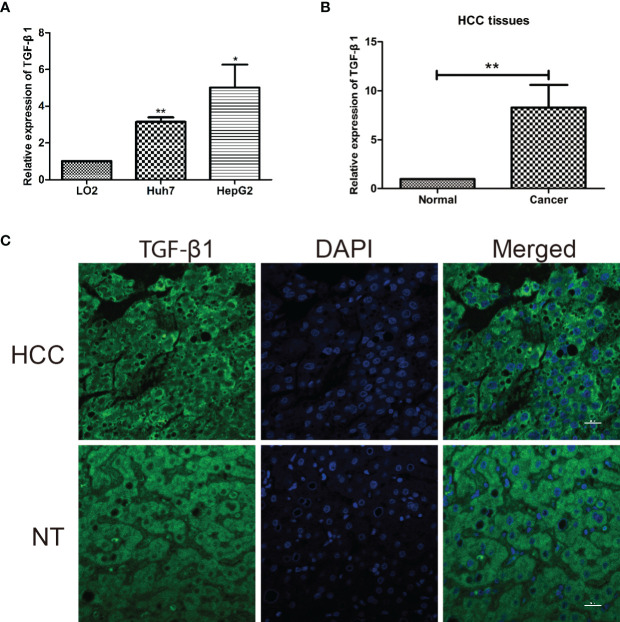
TGF-β1 is highly expressed in HCC cell lines and tissues. **(A)** Relative mRNA expression of TGF-β1 in normal human hepatic cell line (LO2) and two human HCC cell lines (Huh7 and HepG2). TGF-β1 gene expression is shown as the mean ± SD of three independent experiments. **(B)** Relative mRNA expression of TGF-β1 in HCC tissues is significantly higher than that in the paired neighboring non-tumor tissues. TGF-β1 gene expression is shown as the mean ± SD of fifteen tissue pairs. *β-ACTIN* was used as an internal control in RT-qPCR (**p < 0.01, Student’s *t*-test). **(C)** Microscopic images show liver cross-sections of paired para- and cancerous biopsies. TGF-β1 protein level (green) was visualized by indirect immunofluorescence staining, while nuclei DNA (blue) by DAPI staining. The images are representatives from three pairs of HCC and non-tumorous neighboring tissues. HCC, hepatocellular carcinoma; NT, neighboring non-tumorous tissue from the same HCC patient. *P < 0.05.

Finally, in agreement with the above elaborated bioinformatic analyses, molecular and cellular evidence showed higher mRNA expression of TGF-β1 in two human HCC cell lines (Huh7 and HepG2) than in the normal human hepatic cell line (LO2) (**Figure 5A**) and in HCC tissues than in the corresponding paired non-tumorous liver tissues (**Figure 5B**), which was confirmed by immunofluorescent staining at protein level, with TGF-β1 expressed in cytoplasm (**Figure 5C**).

### Involvement of TGF-β1 in Immune-Associated Pathways

**Figure 6:**
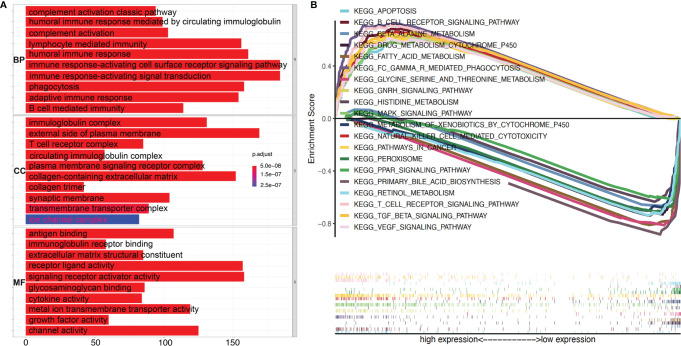
High TGF-β1 expression is positively correlated with active immunity in HCC patients. **(A)** GO (Gene Ontology) analyses using e1071, Parallel, and Preprocesscore packages revealed the involvement of TGF-β1 in activation of various immune-related signal pathways. BP, biological process; CC, cellular component; MF, molecular function. **(B)** Top ten activated and top ten suppressed signal pathways by high TGF-β1 level were presented, respectively, using Gene Set Enrichment Analysis (GSEA) software and KEGG database.

To explore the underlying molecular mechanisms of TGF-β1 in HCC progression and prognosis, Gene Oncology (GO) enrichment and Gene Set Enrichment Analysis (GSEA) were performed against the KEGG database using the extracted TCGA-LIHC RNA-seq data. As shown in **Figure 6A**, most of the significantly enriched biological processes (BP), cellular components (CC), and molecular functions (MF) are immune related, such as humoral immune response, immunoglobulin complex, and antigen binding. These observations from GO enrichment analysis were confirmed by GSEA (**Figure 6B**), which revealed that many pathways were activated by high expression of TGF-β1. Out of the top 50 activated pathways, 10 are enriched in and related to immunity and cancer, as shown in **Figure 6B**, including B cell receptor signal pathway, natural killer cell-mediated cytotoxicity, and T cell receptor signaling pathway. On the other hand, only 12 pathways were found to be significantly downregulated when TGF-β1 was highly expressed in HCC samples, with the first 10 pathways displayed in **Figure 6B**.

**Figure 7:**
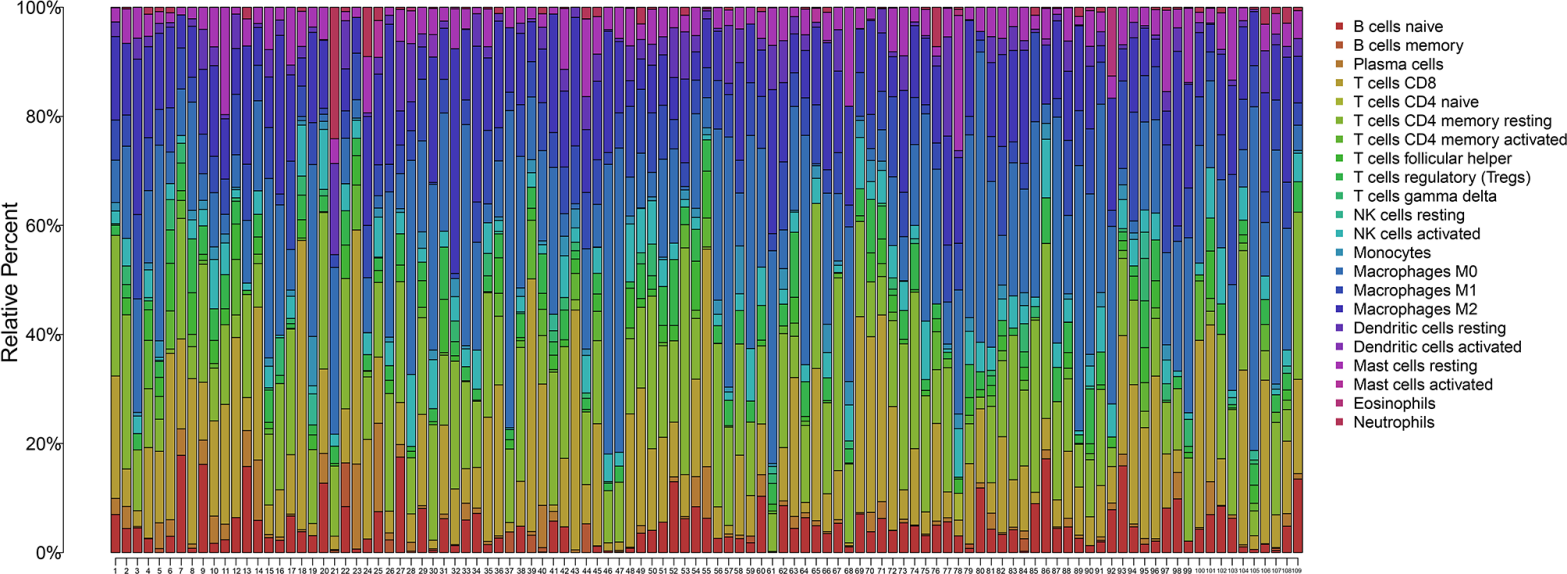
Percentage of the 22 types of immune cells in 109 HCC samples with high TGF-β1 expression using the corrplot package. CIBERSORT tool was used to analyze the data from the 374 HCC patients extracted from TCGA. Data with *p* > 0.05 were filtered out, with 109 samples left as shown in the *x*-axis and **Supplementary Table 2**. The *y*-axis shows the percentage of immune cells in each patient.

**Figure 8:**
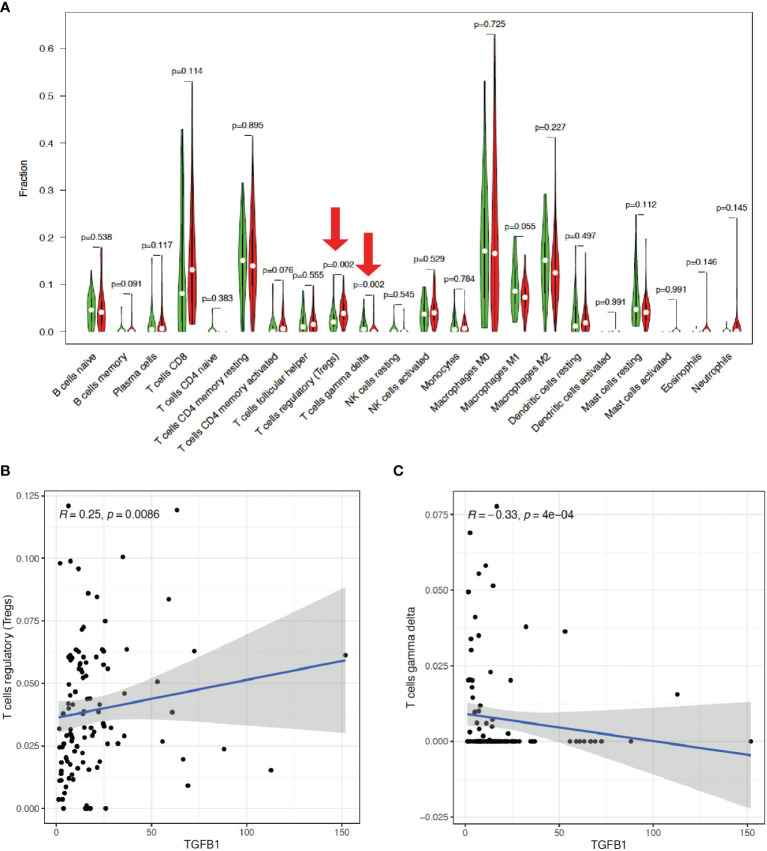
TGF-β1 expression is positively correlated with Tregs, but negatively correlated with γδ T cells. **(A)** Differential analysis of the percentage (*y*-axis) of the 22 types of immune cells (*x*-axis) in HCC patients with high (indicated by red violins) or low (indicated by green violins) TGF-β1 expression by using limma and vioplot packages. The red arrows suggested statistical significance (*p* < 0.05). Correlation analysis of TGF-β1 expression with Tregs **(B)** and γδ T cells **(C)** by using limma, ggplot2, ggpubr, and ggextra packages. One black dot indicates one HCC sample. The blue lines indicate positive **(B)** or negative **(C)** correlation between TGF-β1 expression and immune cell percentage. *R*: correlation coefficient. *p* < 0.05 suggests significant correlation between TGF-β1 expression and immune cell percentage.

Then, the CIBERSORT tool from R was applied to estimate the tumor-infiltrating immune cells (TICs) for the HCC samples based on the RNA-seq datasets. Subsequently, a relationship was observed between the TGF-β1 expression and infiltration of immune cells, with the distribution percentage of 22 immune cell types from 109 HCC patients (**Supplementary Table 2**) shown in **Figure 7**. To further characterize the TICs, differential analysis of the percentage of the 22 types of immune cells in HCC patients revealed that TGF-β1 expression is positively correlated with Tregs, but negatively correlated with γδ T cells (**Figure 8A**), as shown by the blue lines in **Figures 8B, C**.

## Discussion

Liver cancer is one of the most malignant tumors with high morbidity and mortality. The new cases of liver cancer in China each year account for half of the cases worldwide (1). Despite the tremendous progress achieved in surgery, liver transplantation, and radiofrequency ablation, the 5-year survival rate of HCC patients is still rather poor (25). The few and ineffective therapeutic strategies for HCC can be attributed to our poor understanding of its biology. Sorafenib, a tyrosine kinase inhibitor (TKI) to suppress the RAS/RAF/MEK/ERK signal pathway, was approved as a treatment approach for HCC. However, its efficacy is limited by the development of drug resistance and the major neuronal isoform of the pathway; furthermore, BRAF mutation in advanced HCC patients confers a multifocal and/or more aggressive behavior with TKI resistance (26), which altogether urges for more effective alternatives.

Immunotherapy is a novel systemic treatment approach for enhancement of the anti-tumor immune response of HCC patients, which has the potential to reduce tumor recurrence and metastasis (27). TGF-β signaling can promote tumor progression through immunosuppression (6, 28–30), while TGF-β1 can directly suppress immunosurveillance by inhibiting the anti-tumor activity of infiltrating immune cells and subsequently facilitating the recruitment and sustenance of pro-tumoral immune cells to enhance metastatic progression (20).

In the current study, we employed a flora of bioinformatic packages to assess the clinical prognosis value of TGF-β signaling family in the prediction of HCC progression. Thirty-two TGF-β related genes (ACTC1, BMP2, BMP3, BMP4, BMP5, BMP6, BMP7, BMP8A, BMP8B, BMP10, BMP15, GDF1, GDF2, GDF3, GDF5, GDF6, GDF7, GDF9, GDF10, GDF11, GDF15, GDNF, INHA, INHBA, INHBB, LEFTY2, MSTN, NRTN, PSPN, TGFB1, TGFB2, and TGFB3) were subjected to Cox regression analysis and LASSO analysis. The prediction model showed (**Figure 1**) that seven TGF-β family members (BMP2, BMP6, GDF6, GDF7, GDF10, LEFTY2, and TGF-β1) could be potential independent prognostic factors for HCC, which was validated by Kaplan–Meier survival analysis and ROC analysis (**Figure 2**), and further by LASSO analysis and PCA analysis (**Figure 2**). Out of the seven confirmed TGF-β family members, our observation on TGF-β1 is consistently in line with previous studies (31, 32). Considering the high abundance, biological importance, and pathological relevance of TGF-β1 (33–36), we focused on TGF-β1 in this study.

Our global analyses confirmed that TGF-β1 expression is a prognosis factor for HCC progression (**Figure 3**). High TGF-β1 expression was noticed in HCC tissues, with poor survival time (**Figure 4**). In addition, experimental tools revealed that TGF-β1 is highly expressed in HCC tissue and cell lines, compared with the neighboring non-tumorous tissue from the same HCC patients and normal liver cell line (**Figure 5**).

Furthermore, our data showed that TGF-β1 is closely correlated with immune signatures (**Figure 6**), thus regulating the TIME of HCC patients. Among the 22 types of immune cells we analyzed (**Figures 7**, **8**), high TGF-β1 expression in HCC patients is positively correlated with regulatory T cells (Tregs), suggesting that high level of TGF-β1 may activate Tregs to suppress antitumor immunity by inhibiting the killing of HCC cells by antigen-specific CD8+ T cells, leading to poor prognosis (30, 37). On the other hand, the negative connection between TGF-β1 expression in HCC cohorts with γδ T cells (**Figure 8**) may indicate that highly expressed TGF-β1 reduce the number of γδ T cells or suppress the activity of γδ T cells, resulting in more frequent recurrence in HCC patients and poor prognosis (38). Taken together, TGF-β1 can be a promising candidate for immunotherapy in patients with HCC through Tregs and γδ T cells (39–42).

## Conclusion

To summarize, we performed a systemic bioinformatic analysis based on the RNA-seq datasets of HCC cohorts from TCGA and demonstrated that TGF-β1 contributed to HCC progression and poor prognosis *via* regulating Tregs and γδ T cells to modulate tumor immunity. Development of TGF-β1 inhibitors can then potentially help to treat HCC by inhibiting tumor growth and metastasis.

## Data Availability Statement

The original contributions presented in the study are included in the article/**Supplementary Material**. Further inquiries can be directed to the corresponding authors.

## Ethics Statement

All experiments with human tissue samples were reviewed and carried out in accordance with the Declaration of Helsinki and the guidelines of the Ethics Committee of the First Affiliated Hospital of China Medical University (License number 2021–463-2). Informed consents were obtained from all patients. The patients/participants provided their written informed consent to participate in this study.

## Author Contributions

XJ and YLL designed the study. YLL was supported by the grant. XJ, YBL and JL wrote the manuscript. XJ and SZ performed the literature search and collected data for the manuscript. XJ, SZ, NW, and LG analyzed the data and performed the experiments. CS edited the figures and tables. CS and YBL revised the manuscript. All authors contributed to the article and approved the submitted version.

## Funding

This work was supported by the Shenyang Science and Technology Program of 2021 (project number 21-173-9-56) from Shenyang Science and Technology Bureau.

## Conflict of Interest

The authors declare that the research was conducted in the absence of any commercial or financial relationships that could be construed as a potential conflict of interest.

## Publisher’s Note

All claims expressed in this article are solely those of the authors and do not necessarily represent those of their affiliated organizations, or those of the publisher, the editors and the reviewers. Any product that may be evaluated in this article, or claim that may be made by its manufacturer, is not guaranteed or endorsed by the publisher.

## Supplementary Material

Supplementary Figure 1 Confirmation of prognosis model. Lasso regression analysis was implemented to reduce model complexity and prevent over-fitting, and the obtained coefficients as shown in **Figure 1C** was used to calculate the risk score **(A)** and survival time in years **(B)**. **(C)**. Principal Component Analysis (PCA) was applied to further validate the effectiveness of the prognosis model and to display the difference between the two groups (high risk and low risk) using package ggpubr.

## References

[1] Yang JD Hainaut P Gores GJ Amadou A Plymoth A Roberts LR. A Global View of Hepatocellular Carcinoma: Trends, Risk, Prevention and Management. Nat Rev Gastroenterol Hepatol (2019) 16:589–604. doi: 10.1038/s41575-019-0186-y 31439937 PMC6813818

[2] Llovet JM Kelley RK Villanueva A Singal AG Pikarsky E Roayaie S. Hepatocellular Carcinoma. Nat Rev Dis Primers (2021) 7:6. doi: 10.1038/s41572-020-00240-3 33479224 PMC13537433

[3] Liu Y Guo J Huang L. Modulation of Tumor Microenvironment for Immunotherapy: Focus on Nanomaterial-Based Strategies. Theranostics (2020) 10:3099–117. doi: 10.7150/thno.42998 PMC7053194 32194857

[4] Pinato DJ Guerra N Fessas P Murphy R Mineo T Mauri FA. Immune-Based Therapies for Hepatocellular Carcinoma. Oncogene (2020) 39:3620–37. doi: 10.1038/s41388-020-1249-9 PMC7190571 32157213

[5] Hegde PS Chen DS. Top 10 Challenges in Cancer Immunotherapy. Immunity (2020) 52:17–35. doi: 10.1016/j.immuni.2019.12.011 31940268

[6] Batlle E Massague J. Transforming Growth Factor-Beta Signaling in Immunity and Cancer. Immunity (2019) 50:924–40. doi: 10.1016/j.immuni.2019.03.024 PMC7507121 30995507

[7] Hinck AP Mueller TD Springer TA. Structural Biology and Evolution of the TGF-Beta Family. Cold Spring Harb Perspect Biol (2016) 8:a022103. doi: 10.1101/cshperspect.a022103 27638177 PMC5131774

[8] de Caestecker MP Piek E Roberts AB. Role of Transforming Growth Factor-Beta Signaling in Cancer. J Natl Cancer Inst (2000) 92:1388–402. doi: 10.1093/jnci/92.17.1388 10974075

[9] Derynck R Turley SJ Akhurst RJ. TGFbeta Biology in Cancer Progression and Immunotherapy. Nat Rev Clin Oncol (2021) 18:9–34. doi: 10.1038/s41571-020-0403-1 32710082 PMC9721352

[10] Gonzalez-Sanchez E Vaquero J Fernandez-Barrena MG Lasarte JJ Avila MA Sarobe P. The TGF-Beta Pathway: A Pharmacological Target in Hepatocellular Carcinoma? Cancers (Basel) (2021) 13(13):3248. doi: 10.3390/cancers13133248 34209646 PMC8268320

[11] Massague J. TGFbeta in Cancer. Cell (2008) 134:215–30. doi: 10.1016/j.cell.2008.07.001 PMC3512574 18662538

[12] Seoane J Gomis RR. TGF-Beta Family Signaling in Tumor Suppression and Cancer Progression. Cold Spring Harb Perspect Biol (2017) 9:a022277. doi: 10.1101/cshperspect.a022277 28246180 PMC5710110

[13] Gupta DK Singh N Sahu DK. TGF-Beta Mediated Crosstalk Between Malignant Hepatocyte and Tumor Microenvironment in Hepatocellular Carcinoma. Cancer Growth Metastas (2014) 7:1–8. doi: 10.4137/CGM.S14205 PMC3988670 24741325

[14] Baghban R Roshangar L Jahanban-Esfahlan R Seidi K Ebrahimi-Kalan A Jaymand M. Tumor Microenvironment Complexity and Therapeutic Implications at a Glance. Cell Commun Signal (2020) 18:59. doi: 10.1186/s12964-020-0530-4 32264958 PMC7140346

[15] Wang M Zhao J Zhang L Wei F Lian Y Wu Y. Role of Tumor Microenvironment in Tumorigenesis. J Cancer (2017) 8:761–73. doi: 10.7150/jca.17648 PMC5381164 28382138

[16] Roth GS Decaens T. Liver Immunotolerance and Hepatocellular Carcinoma: Patho-Physiological Mechanisms and Therapeutic Perspectives. Eur J Cancer (2017) 87:101–12. doi: 10.1016/j.ejca.2017.10.010 29145036

[17] Mao X Xu J Wang W Liang C Hua J Liu J. Crosstalk Between Cancer-Associated Fibroblasts and Immune Cells in the Tumor Microenvironment: New Findings and Future Perspectives. Mol Cancer (2021) 20:131. doi: 10.1186/s12943-021-01428-1 34635121 PMC8504100

[18] Wang Y Zhang T Sun M Ji X Xie M Huang W. Therapeutic Values of Myeloid-Derived Suppressor Cells in Hepatocellular Carcinoma: Facts and Hopes. Cancers (Basel) (2021) 13:5127. doi: 10.3390/cancers13205127 34680276 PMC8534227

[19] Zhang Y Lazarus J Steele NG Yan W Lee HJ Nwosu ZC. Regulatory T-Cell Depletion Alters the Tumor Microenvironment and Accelerates Pancreatic Carcinogenesis. Cancer Discov (2020) 10:422–39. doi: 10.1158/2159-8290.CD-19-0958 PMC7224338 31911451

[20] Angioni R Sanchez-Rodriguez R Viola A Molon B. TGF-Beta in Cancer: Metabolic Driver of the Tolerogenic Crosstalk in the Tumor Microenvironment. Cancers (Basel) (2021) 13:401. doi: 10.3390/cancers13030401 33499083 PMC7865468

[21] Stuelten CH Zhang YE. Transforming Growth Factor-Beta: An Agent of Change in the Tumor Microenvironment. Front Cell Dev Biol (2021) 9:764727. doi: 10.3389/fcell.2021.764727 34712672 PMC8545984

[22] Morikawa M Derynck R Miyazono K. TGF-Beta and the TGF-Beta Family: Context-Dependent Roles in Cell and Tissue Physiology. Cold Spring Harb Perspect Biol (2016) 8:a021873. doi: 10.1101/cshperspect.a021873 27141051 PMC4852809

[23] Lin LF Doherty DH Lile JD Bektesh S Collins F. GDNF: A Glial Cell Line-Derived Neurotrophic Factor for Midbrain Dopaminergic Neurons. Science (1993) 260:1130–2. doi: 10.1126/science.8493557 8493557

[24] Sabbadini F Bertolini M De Matteis S Mangiameli D Contarelli S Pietrobono S. The Multifaceted Role of TGF-Beta in Gastrointestinal Tumors. Cancers (Basel) (2021) 13:3960. doi: 10.3390/cancers13163960 34439114 PMC8391793

[25] Allemani C Weir HK Carreira H Harewood R Spika D Wang XS. Global Surveillance of Cancer Survival 1995-2009: Analysis of Individual Data for 25,676,887 Patients From 279 Population-Based Registries in 67 Countries (CONCORD-2). Lancet (2015) 385:977–1010. doi: 10.1016/S0140-6736(14)62038-9 25467588 PMC4588097

[26] Gnoni A Licchetta A Memeo R Argentiero A Solimando AG Longo V. Role of BRAF in Hepatocellular Carcinoma: A Rationale for Future Targeted Cancer Therapies. Medicina (Kaunas) (2019) 55(12):754. doi: 10.3390/medicina55120754 PMC6956203 31766556

[27] Bo XW Sun LP Yu SY Xu HX. Thermal Ablation and Immunotherapy for Hepatocellular Carcinoma: Recent Advances and Future Directions. World J Gastrointest Oncol (2021) 13:1397–411. doi: 10.4251/wjgo.v13.i10.1397 PMC8529921 34721773

[28] Tauriello DVF Palomo-Ponce S Stork D Berenguer-Llergo A Badia-Ramentol J Iglesias M. TGFbeta Drives Immune Evasion in Genetically Reconstituted Colon Cancer Metastasis. Nature (2018) 554:538–43. doi: 10.1038/nature25492 29443964

[29] Mariathasan S Turley SJ Nickles D Castiglioni A Yuen K Wang Y. TGFbeta Attenuates Tumour Response to PD-L1 Blockade by Contributing to Exclusion of T Cells. Nature (2018) 554:544–8. doi: 10.1038/nature25501 PMC6028240 29443960

[30] Budhu S Schaer DA Li Y Toledo-Crow R Panageas K Yang X. Blockade of Surface-Bound TGF-Beta on Regulatory T Cells Abrogates Suppression of Effector T Cell Function in the Tumor Microenvironment. Sci Signal (2017) 10(494). doi: 10.1126/scisignal.aak9702 PMC5851440 28851824

[31] Lin TH Shao YY Chan SY Huang CY Hsu CH Cheng AL. High Serum Transforming Growth Factor-Beta1 Levels Predict Outcome in Hepatocellular Carcinoma Patients Treated With Sorafenib. Clin Cancer Res (2015) 21:3678–84. doi: 10.1158/1078-0432.CCR-14-1954 25977342

[32] Ji F Fu SJ Shen SL Zhang LJ Cao QH Li SQ. The Prognostic Value of Combined TGF-Beta1 and ELF in Hepatocellular Carcinoma. BMC Cancer (2015) 15:116. doi: 10.1186/s12885-015-1127-y 25880619 PMC4359586

[33] Poniatowski LA Wojdasiewicz P Gasik R Szukiewicz D. Transforming Growth Factor Beta Family: Insight Into the Role of Growth Factors in Regulation of Fracture Healing Biology and Potential Clinical Applications. Mediators Inflamm (2015) 2015:137823. doi: 10.1155/2015/137823 25709154 PMC4325469

[34] Kubiczkova L Sedlarikova L Hajek R Sevcikova S. TGF-Beta - an Excellent Servant But a Bad Master. J Transl Med (2012) 10:183. doi: 10.1186/1479-5876-10-183 22943793 PMC3494542

[35] Hayashi H Sakai T. Biological Significance of Local TGF-Beta Activation in Liver Diseases. Front Physiol (2012) 3:12. doi: 10.3389/fphys.2012.00012 22363291 PMC3277268

[36] Lin RL Zhao LJ. Mechanistic Basis and Clinical Relevance of the Role of Transforming Growth Factor-Beta in Cancer. Cancer Biol Med (2015) 12:385–93. doi: 10.7497/j.issn.2095-3941.2015.0015 PMC4706525 26779375

[37] Wang Y Liu T Tang W Deng B Chen Y Zhu J. Hepatocellular Carcinoma Cells Induce Regulatory T Cells and Lead to Poor Prognosis *via* Production of Transforming Growth Factor-Beta1. Cell Physiol Biochem (2016) 38:306–18. doi: 10.1159/000438631 26799063

[38] Cai XY Wang JX Yi Y He HW Ni XC Zhou J. Low Counts of Gammadelta T Cells in Peritumoral Liver Tissue are Related to More Frequent Recurrence in Patients With Hepatocellular Carcinoma After Curative Resection. Asian Pac J Cancer Prev (2014) 15:775–80. doi: 10.7314/apjcp.2014.15.2.775 24568494

[39] Tian W Ma J Shi R Ren C He J Zhao H. Gammadelta T Cell-Mediated Individualized Immunotherapy for Hepatocellular Carcinoma Considering Clinicopathological Characteristics and Immunosuppressive Factors. Oncol Lett (2018) 15:5433–42. doi: 10.3892/ol.2018.8026 PMC5840521 29552184

[40] Kabelitz D Serrano R Kouakanou L Peters C Kalyan S. Cancer Immunotherapy With Gammadelta T Cells: Many Paths Ahead of Us. Cell Mol Immunol (2020) 17:925–39. doi: 10.1038/s41423-020-0504-x PMC7609273 32699351

[41] Langhans B Nischalke HD Kramer B Dold L Lutz P Mohr R. Role of Regulatory T Cells and Checkpoint Inhibition in Hepatocellular Carcinoma. Cancer Immunol Immunother (2019) 68:2055–66. doi: 10.1007/s00262-019-02427-4 PMC11028391 31724091

[42] Bozward AG Warricker F Oo YH Khakoo SI. Natural Killer Cells and Regulatory T Cells Cross Talk in Hepatocellular Carcinoma: Exploring Therapeutic Options for the Next Decade. Front Immunol (2021) 12:643310. doi: 10.3389/fimmu.2021.643310 33995362 PMC8120158

