# Supplementary figures and images for: High Expression of TGF-β1 Contributes to Hepatocellular Carcinoma Prognosis via Regulating Tumor Immunity
Source: Front Oncol. 2022 Apr 25;12:861601. doi: 10.3389/fonc.2022.861601 (PMC9082360; doi:10.3389/fonc.2022.861601)

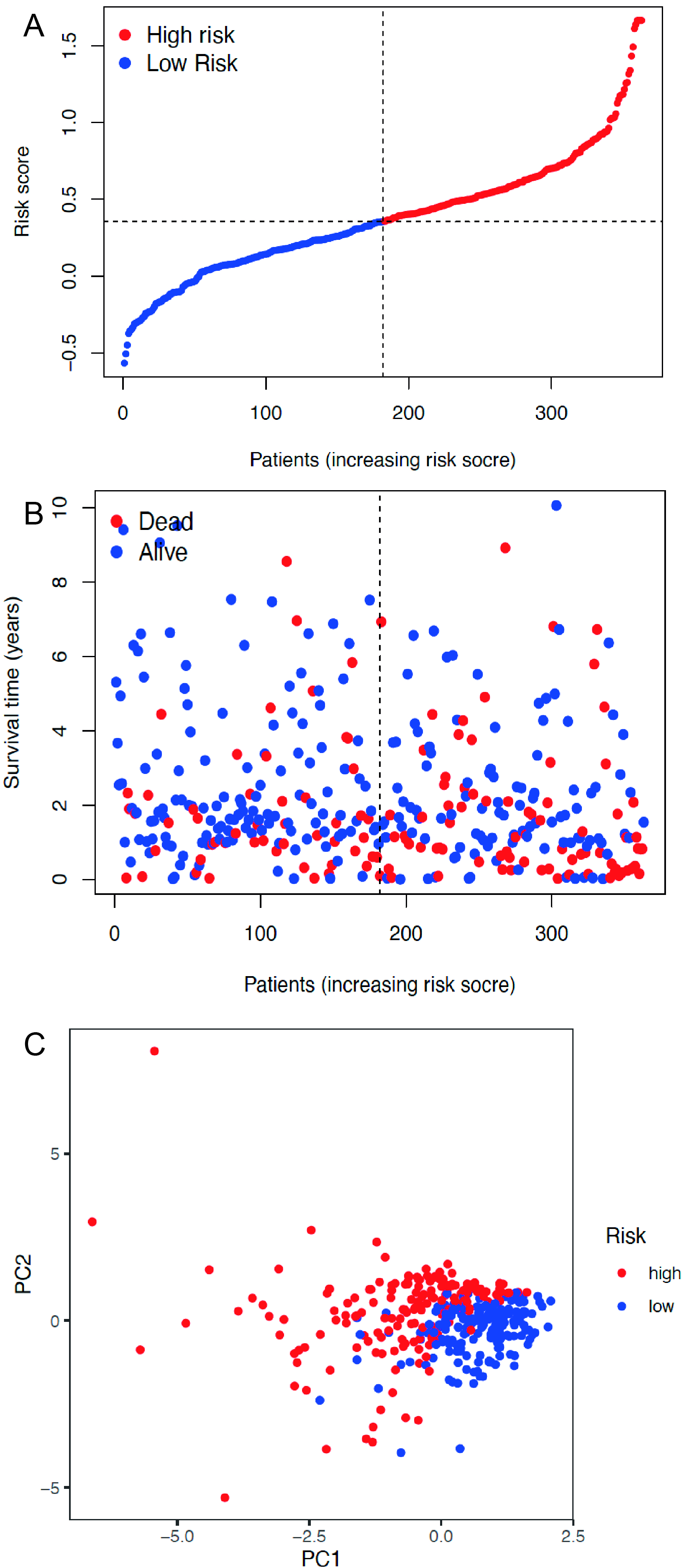

Supplement: Supplementary Figure 1 — Confirmation of prognosis model. Lasso regression analysis was implemented to reduce model complexity and prevent over-fitting, and the obtained coefficients as shown in Figure 1C was used to calculate the risk score (A) and survival time in years (B). (C). Principal Component Analysis (PCA) was applied to further validate the effectiveness of the prognosis model and to display the difference between the two groups (high risk and low risk) using package ggpubr. [file Image_1.jpeg]
